# Vertical distribution of zooplankton: density dependence and evidence for an ideal free distribution with costs

**DOI:** 10.1186/1741-7007-3-10

**Published:** 2005-04-06

**Authors:** Winfried Lampert

**Affiliations:** 1 Max Planck Institute for Limnology, Department of Physiological Ecology, Postfach 165, 24302 Plön, Germany

## Abstract

**Background:**

In lakes with a deep-water algal maximum, herbivorous zooplankton are faced with a trade-off between high temperature but low food availability in the surface layers and low temperature but sufficient food in deep layers. It has been suggested that zooplankton (*Daphnia*) faced with this trade-off distribute vertically according to an "Ideal Free Distribution (IFD) with Costs". An experiment has been designed to test the density (competition) dependence of the vertical distribution as this is a basic assumption of IFD theory.

**Results:**

Experiments were performed in large, indoor mesocosms (Plankton Towers) with a temperature gradient of 10°C and a deep-water algal maximum established below the thermocline. As expected, Daphnia aggregated at the interface between the two different habitats when their density was low. The distribution spread asymmetrically towards the algal maximum when the density increased until 80 % of the population dwelled in the cool, food-rich layers at high densities. Small individuals stayed higher in the water column than large ones, which conformed with the model for unequal competitors.

**Conclusion:**

The *Daphnia *distribution mimics the predictions of an IFD with costs model. This concept is useful for the analysis of zooplankton distributions under a large suite of environmental conditions shaping habitat suitability. Fish predation causing diel vertical migrations can be incorporated as additional costs. This is important as the vertical location of grazing zooplankton in a lake affects phytoplankton production and species composition, i.e. ecosystem function.

## Background

The water column in a stratified lake provides vertical gradients of habitat qualities for zooplankton. Surface layers (epilimnion) and deep layers (hypolimnion) separated by a strong temperature gradient (thermocline) differ very much with respect to temperature, light, food availability and predation risk. Although zooplankton are defined as "floating" in the water column, their populations show distinct horizontal and vertical distribution patterns [1]. At least the vertical distribution is the result of active habitat choice. Diel vertical migration is a striking example of habitat shift in response to changing suitability. Large zooplankton leave the warm, lighted and often food-rich epilimnion during the day to dwell in the cold, dark hypolimnion where food may be of low quantity and poor quality, in order to avoid predation by visually hunting predators (fish). They return to the surface layers at night when the predation risk is small [2]. Numerous studies have shown that this pattern is influenced by food conditions [3-5] and temperature gradient [6,7].

However, there is increasing evidence that the vertical distribution of algal food for herbivorous zooplankton is not always "typical". In particular, oligotrophic and mesotrophic lakes often exhibit a deep-water algal maximum, i.e. highest algal densities are not found in the epilimnion but in the upper hypolimnion, below the thermocline [8-10]. Zooplankton then face a trade-off between high temperature (fast development) but poor food (low reproductive potential) in the epilimnion and low temperature but high food availability in the hypolimnion [11]. In the absence of visual predation risk (e. g. in lakes with scarce populations of planktivorous fish or at night), herbivorous zooplankton should, therefore, distribute vertically so as to optimise the fitness gain depending on the temperature and food gradient. In fact, it has been observed that zooplankton forced to deep layers by fish predation during the day return to the warm epilimnion at night even if their food is more abundant in the hypolimnion [10,12].

It has been suggested [11] and evidence has been given that the vertical distribution of herbivorous zooplankton (*Daphnia*) faced with the trade-off follows an "Ideal Free Distribution (IFD) with Costs" [13]. However, [11] placed a question mark after the title of their paper as, although their experimental results resembled an IFD with costs, the mechanism was not yet clear. Since then, several new experiments have shown that some of the assumptions underlying the IFD model [14] are fulfilled in the system. (1) *Daphnia *are "ideal" organisms as they select the habitat best suited to their survival and reproduction. The proportion of individuals dwelling in a particular habitat is correlated to the relative fitness gain in that habitat [15]. (2) *Daphnia *are also "free" to choose the habitat. Every habitat is equally accessible to all members of the population. The observed distribution is dynamic, i. e. it is the equilibrium result of individuals moving randomly up and down and allocating a proportion of their time to a certain habitat [16]. (3) The IFD model assumes that all individuals are equal. Being members of a clone, *Daphnia *are genetically equal, but differing in size they may not be metabolically equal. Small differences in depth distributions of different size classes have been found [17], but only during the day (i.e. probably related to predation risk). This problem can be overcome by looking at the response of individual size classes separately.

Although several assumptions underlying the IFD have now been tested in this system, the one that is probably most important has not. The IFD model strongly requires that habitat suitability is density dependent. Unfortunately, information on density dependence of *Daphnia *distributions so far is scarce, and only a weak effect has been found [18]. The purpose of this study was to measure the density dependence of the vertical distribution. The study was designed to test the following hypotheses. (1) The vertical distribution becomes broader as *Daphnia *density increases. (2) As density effects are related to food availability, but not to temperature, *Daphnia *will, at higher densities, spread asymmetrically to deeper layers in order to exploit the deep-water algal maximum better. (3) Differences in the depth distributions of differently sized *Daphnia *can be explained by intraspecific competition.

## Results

**Figure 1:**
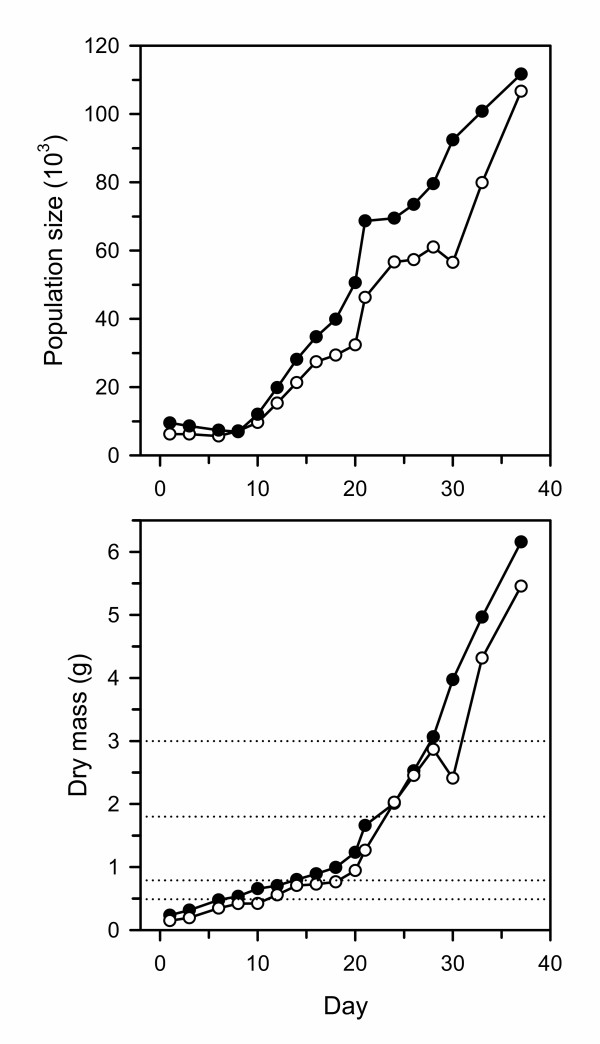
Population size and total dry mass of *D. pulicaria *in the two mesocosms (closed symbols = tower 1; open symbols = tower 2). Dotted lines delineate the biomass groups used for the analysis.

*Daphnia *populations in both columns started to grow after a short lag-phase (Fig. 1). Total biomass increased steadily from the very beginning indicating that individuals accumulated body mass before they started reproducing. Although the absolute numbers of *Daphnia *differed between the two towers, possibly due to small differences in the inoculum, the population growth pattern was similar. The differences between the two towers were due to large numbers of very small individuals in tower 1. As the small individuals contribute less to the total biomass, differences were less pronounced for biomasses than for numbers.

**Figure 2:**
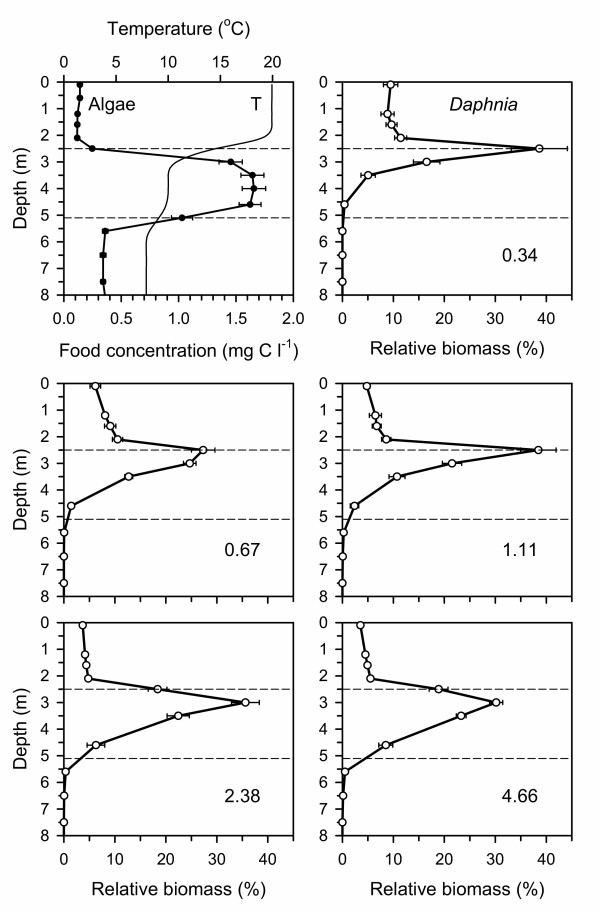
Upper left: Mean vertical gradients of temperature (thin line) and algal concentration (closed circles) during the experiment. Thermal variation was too small to be depicted in this graph. Other panels: Vertical biomass distributions of the total population of *D. pulicaria *at increasing densities. Numbers in different panels indicate the mean dry biomass in g per tower in the five total biomass groups (both towers combined). Broken lines delimit the zone with the algal maximum. Error bars: ± 1 s.e.

The thermocline was stable at 2.5 m, and the deep-water algal maximum was maintained throughout the experimental period with little variation (Fig. 2). Particle concentration in the epilimnion varied between 0.05 and 0.19 mg C L^-1 ^with a trend to higher particle concentrations towards the end of the experiment. There was a significant linear relationship (n = 33, r^2 ^= 0.624) between the particle concentration in the epilimnion (P_epi_) and the log-transformed *Daphnia *biomass (B_max_) in the algal maximum (P_epi _= 0.059 log(B_max_) + 0.129).

**Figure 3:**
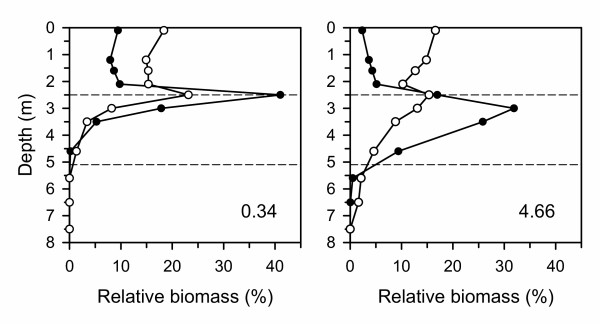
Comparison of the mean vertical biomass distributions of the smallest (open circles) and the largest (closed circles) size groups of *D. pulicaria *at the lowest (left) and the highest (right) densities (cf. Fig. 2).

Vertical distributions were assigned to five classes (cf. Fig. 1 and Methods) of increasing total *Daphnia *biomass (g dry mass per tower), and the mean distributions are shown in Fig. 2. The vertical distribution changed with biomass density. At low population densities, biomass peaked sharply at the thermocline, but the distribution spread out when the total biomass increased (Fig. 2). The peak moved below the thermocline at the time when total *Daphnia *biomass was highest, and the distribution broadened further. However, there were differences in the distributions of different size classes of *Daphnia *(Fig. 3). Small size classes tended to stay higher in the water column than large ones, regardless of the total *Daphnia *biomass, although their distribution spread out, too, even below the algal maximum.

The results of visual inspection of the vertical distributions are confirmed by Principal Component Analysis (PCA, Table 1). The first three principal components (PC1–PC3) explain more than 95 % of the variation. PC1 contrasts the biomass proportions at the thermocline (port 6, 2.5 m) with the proportions at ports 7 and 8, i.e. the algal maximum. Hence it depicts the downward spreading of the distribution. PC2 contrasts the proportions at the thermocline with those in the epilimnion. Finally, PC3 depicts the distributional shifts within the algal maximum, but its contribution is very small so it is not considered.

The ANOVA on the factor scores of PC1 shows significant influences of total biomass (Mass) and *Daphnia *size class (Size) on the vertical distribution, and a significant interaction between these two factors (Table 2). This confirms the density dependence of the distribution as well as the different responses of size groups to total density. Total density had no significant effect on the factor scores of PC2, but there were significant effects of Size and Tower, a significant Mass × Tower interaction, and a marginally significant Size × Tower interaction. This shows that PC2 (contrast between thermocline and epilimnion) is mainly influenced by *Daphnia *size (cf. Fig. 3). The significant Mass × Tower and Size × Tower interactions suggest that the tower effect on PC2 is a consequence of differences in size composition of the populations.

**Figure 4:**
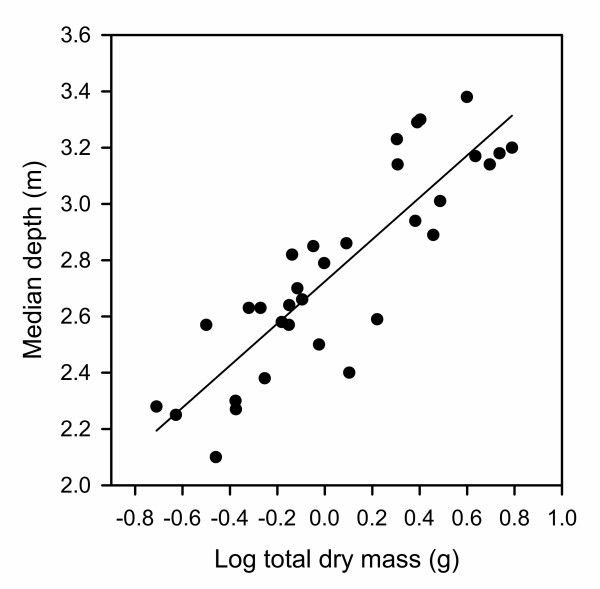
Relationship between the total biomass of *D. pulicaria *per tower and the median depth of the biomass distribution (both towers combined). Each point represents a single vertical profile.

The descriptive analysis of the shape of the vertical distributions was followed by more quantitative approaches with ungrouped samples. Figure 4 depicts the positive relationship between the median of the *Daphnia *depth distribution and total density. The higher the *Daphnia *biomass per tower, the deeper the median. A linear regression between median depth (MD) and the log-transformed biomass (B) is significant (MD = 0.746 log(B) + 2.72, n = 33, r^2 ^= 0.754).

**Figure 5:**
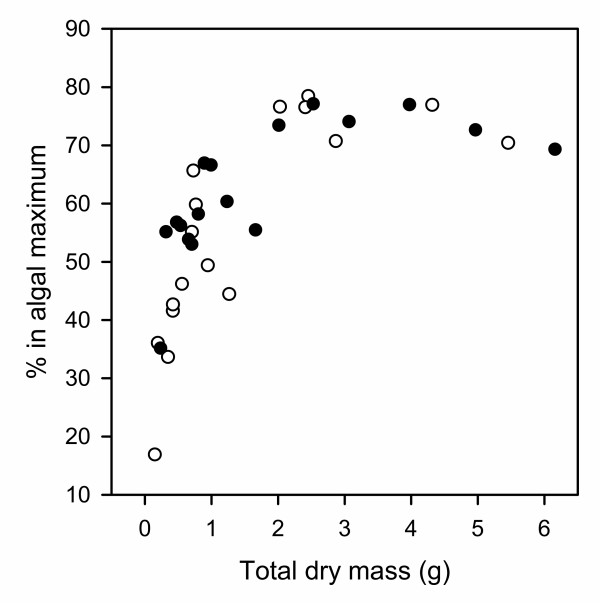
Proportion of the total *Daphnia *biomass dwelling in the algal maximum (cf. Fig. 2) in relation to total density (g per tower). Symbols indicate the two towers.

**Figure 6:**
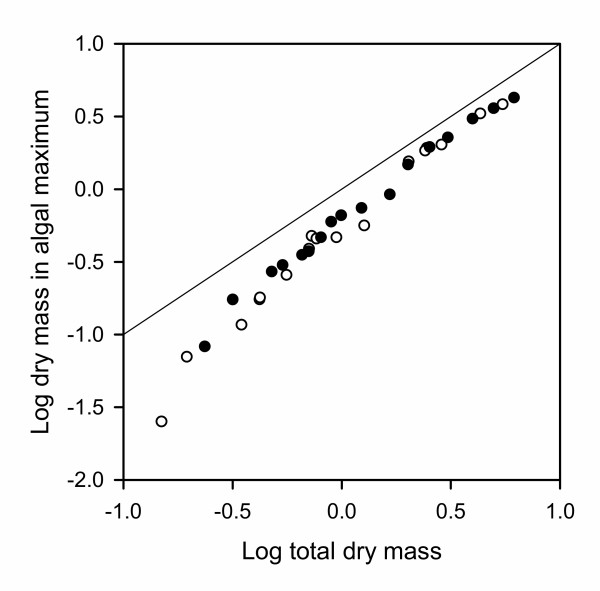
Relation between the total mass of *Daphnia *per tower and in the algal maximum. Symbols indicate the two mesocosms. Line of equality indicated.

The proportion of the total population (in terms of biomass) dwelling within the algal maximum increased strongly with total density, but levelled off at ca. 80 % above a total *Daphnia *biomass of 3 g per tower (Fig. 5). The relationship between the log-transformed biomass in the total tower and the log-transformed biomass in the algal maximum was linear (Fig. 6). In order to improve homoscedasticity and to obtain a conservative estimate of the slope, the lowest biomass value (initial sample of tower 2) was excluded from the analysis. There was no difference between towers so the values were pooled. The slope of the resulting regression (Table 3) is significantly larger than 1 (d.f. = 32, F = 57.3, p < 0.001), i.e. with increasing total biomass, disproportionally more *Daphnia *dwelled in the algal maximum.

As *Daphnia *size affected the response to total biomass (Mass × Size interaction for PC1), the overall regression was broken down to size classes (Table 3). There was no significant difference between the slopes of the individual regressions, but the intercepts were different and showed a clear trend, increasing with *Daphnia *size class. Consequently, all size classes moved toward the algal maximum with increasing total density, but large *Daphnia *moved deeper than small ones.

**Figure 7:**
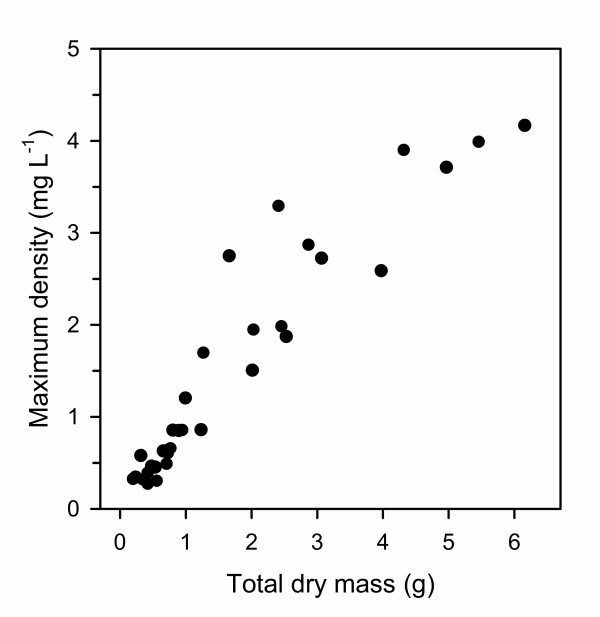
Maximum density of *Daphnia *in a vertical profile (peak) in dependence on the total biomass per tower.

Maximum (peak) *Daphnia *density in each vertical profile increased with total biomass, but the relationship was not linear (Fig. 7). Rather, an upper limit between 4 and 5 mg L^-1 ^(approximately 80–90 individuals L^-1^) seems to be approached when the biomass per tower reached high values. This is direct evidence for density dependence of the distribution, and it provides an estimate of the maximum density *Daphnia pulicaria *can tolerate under these conditions.

## Discussion

Vertical distribution and diel vertical migrations of zooplankton have been studied for a long time [19] but are still not fully understood. This study fills an important gap by revealing an additional mechanism (density dependence) controlling the vertical distribution of zooplankton. Density dependence is a basic determinant of IFD theory [14,20], and the results of this study conform to the theory. Considering that the conditions in this system differ strongly from the original IFD model, this shows that the concept is rather robust, even if it only "mimics" an IFD [21].

For simplicity, IFD models have been developed for discrete patches of habitat suitability, where the differences in habitat suitability were caused solely by biotic factors (food availability, competition). Such a situation has been tested when [22] found that *Daphnia *distributions between food patches complied with IFD predictions. The "IFD with Costs" model [13] considers in addition the effect of abiotic factors. This model is more appropriate for the present situation as higher food availability is linked to higher costs (low temperature). But in contrast to water currents, as in [13], temperature does not only affect the net energy gain; it also limits the speed of development, which is a particularly important factor in parthenogenetic animals with continuous reproduction such as *Daphnia*. Consequently, ignoring interference from competitors, the basic habitat suitability [14] is the result of the total fitness gain, not just resource input. Also, vertical habitat structure in a lake does not represent discrete patches but changes over gradients, which may not be a problem as IFD models have been shown to be applicable for environmental gradients [23]. However, the vertical temperature gradient in a lake is not smooth. The thermal structure rather creates two different habitats (epilimnion and hypolimnion) connected by a steep gradient (thermocline). We may consider this situation as two habitat patches with some overlap. Because of the trade-off, it is possible that the optimum habitat suitability is in the overlap zone, i.e. in the thermocline. In fact, earlier experiments with similar conditions (10°C temperature gradient) [11,17] resulted in pronounced distribution peaks at the thermocline, very much like the distributions found in this study at low *Daphnia *densities. Population densities in the earlier studies were not controlled, but a re-analysis of the raw data of [11] showed that they ranged from 0.3–2.8 g dry mass per tower, which is in the lower range of this study.

The sharp aggregation in the thermocline can be profitable as long as density effects are negligible. *Daphnia *are not perfectly "ideal" organisms since they do not have complete knowledge about the suitability of all habitats. Hence they need to move around and sample the habitats. The nature of the trade-off may also require that they spend some time in both habitats. It has been demonstrated [16] that the vertical distribution is dynamic, i.e. it is the equilibrium result of random movements and different time allocations of the individuals. Since food availability and temperature are uniform in the hypolimnion as well as in the epilimnion, swimming longer distances up and down does not pay off. The steep environmental gradient provides the opportunity to access sufficient food and high temperatures within a short distance, as long as interference from competitors is low.

This changes at high densities when competitive interactions become important. The negative effect of high competitor densities inevitably leads to a broadening of the distribution. There are two mechanisms of competitive interactions in filter-feeding *Daphnia*. Interference may cause a reduction of food intake when encounters between individuals become too frequent and the continuous filtering process is disturbed [24,25]. Exploitation reduces the food concentration through joint filtering activity. The latter process can only be locally important in the tower system as algal concentrations in the hypolimnion were maintained at approximately 1.5 mg C L^-1^, which is above the "incipient limiting concentration" where the feeding rate of *Daphnia *becomes independent of the food concentration [26]. Only at the upper edge of the food maximum (at the thermocline) may exploitation competition play a role due to the high grazing pressure on algae entering the thermocline from below due to small-scale turbulence. The sharp upper edge of the algal distribution is probably a result of grazing.

Both types of interactions among *Daphnia *lead to reduced food uptake. On the other hand, density has little negative influence on the temperature effect (eventually through enhanced metabolic activity). This must cause an asymmetric response of the *Daphnia*. Spreading into the thermally homogeneous food maximum will have a positive effect since avoiding competitors means obtaining more food with no additional temperature costs, except the additional costs of swimming, which are small [27]. Spreading towards the surface will have little or no positive effect as energy input is limited by the low algal concentration, not by competitors. The experimental results conform well with this prediction. There is a significant effect on the shape of the distribution at high *Daphnia *densities. The vertical distribution becomes broader and shifts into the algal maximum. Although food availability and temperature are identical over the whole hypolimnion, *Daphnia *do not distribute homogeneously, but the distribution is skewed towards the upper edge. Again, this points to the fact that the optimum habitat at low densities is near the thermocline where access to warm water and food is easiest. The *Daphnia *distribution follows a gradient of costs (to reach warm water), not resources. The shift can be quantified by the positive relationship between *Daphnia *density and median depth, and the increasing proportion of *Daphnia *dwelling within the algal maximum. The density effect is as strong as the effect of increasing hypolimetic temperature [11].

One disadvantage of the experimental design is the correlation of *Daphnia *density with time. It may be argued that the downward spread of the distribution is the result of some unknown factor changing with time. For example, the epilimnetic habitat suitability may have become worse if the epilimnetic food conditions deteriorated with time. Measurements do not support this caveat. Although the mean particle volume was equivalent to about 0.1 mg C L^-1^, there were nearly no intact *Scenedesmus *cells present. The measured particles were small and probably comprised material (e.g. empty cell walls) that had been recycled by *Daphnia*; thus the food quality was low. In fact, the epilimnetic particle concentration even tended to increase slightly towards the end of the experiment, which if the food were good should have resulted in an upward shift. The significant relationship between particle volume in the epilimnion and total *Daphnia *biomass rather suggests that the increase in epilimnetic particles was due to *Daphnia *feeding in deep layers and defecating in the epilimnion [16].

A final argument supporting the view that *Daphnia *density is the driving force for the downward shift comes from the estimate of maximum local densities. The distribution is not shaped directly by the total biomass of *Daphnia *in the tower, but by the local density at each depth. The maximum local density tends to reach a plateau at 4–5 mg L^-1^, which is an estimate of the upper limit of density tolerance under the given food conditions.

IFD theory assumes that all individuals are equally good competitors [14], but there is little evidence to support this [21]. In nature, individuals will have different competitive abilities, and this requires modified concepts [28]. The concept of unequal competitors offers a large range of possible distributions [28], and only a special case yields a distribution with equal proportions of different competitors that "mimics" an IFD [29]. Belonging to a single clone, the *Daphnia *in this experiment are at least genetically equal, but they differ in size. Under constant conditions, large *Daphnia *are considered to be competitively superior to small ones. There is mechanistic evidence for this relationship on the level of interspecific comparisons [30,31]. Mechanistic support for a similar intraspecific relationship comes from an unpublished laboratory study by C. Kreutzer and M. Boersma. They found that large individuals of *D. pulicaria *had lower food thresholds for growth than small ones, i.e. were competitively superior. Evidence for intraspecific competition is often inferred from segregation of different size classes in field studies [32,33], but the interpretation is hampered by the fact that fish predation has a similar size-selective effect, hence the differing depth distributions of small and large individuals may be due to a trade-off between their ability to exploit resources and their susceptibility to predation [34]. However, fish predation and any cue for the presence of fish was excluded in this tower experiment.

The experimental results are consistent with the predictions of the model assuming the competitive superiority of large individuals. A greater proportion of small *Daphnia *than of large ones is present in the epilimnion (Fig. 3). The distributions at low *Daphnia *densities are very similar to those found under the same conditions by [17], but the differences in the shapes of the distributions become more pronounced at higher *Daphnia *densities (increased competition). Small *Daphnia *spread out in both directions, even into the region below the algal maximum (where food is still more abundant than in the epilimnion). The relatively larger fraction of small *Daphnia *in the epilimnion suggests that temperature is a more important factor for small than for large individuals. The smallest size class in this study contained varying proportions of neonates that are probably not so dependent on high food concentrations during their first hours of life, as they still carry some yolk left from the embryonic stage. Small-scale experiments in stratified columns designed to study individual swimming behavior showed, in fact, that neonates crossed the thermocline much less frequently than adults [35].

If the relative competitive ability of unequal competitors does not change in different habitats, the distribution of competitors can be replaced by the distribution of the sum of competitive abilities. Consequently, there are numerous possible combinations of phenotypes in the different habitats [28]. Nevertheless, it is highly probable that a solution is met where the proportions of different phenotypes are identical in all habitats (for review see [29]). The results of this experiment conform with predictions for constant relative competitive abilities in the different habitats. The proportion of individuals dwelling in the algal maximum increases with *Daphnia *densities in all four size classes (Table 3) since the regression slopes are all above unity. There is a significant trend in the elevations, indicating that the size classes differ in their mean depth. However, the slopes of the regressions do not differ, i.e. the relative proportions of the size classes in the algal maximum remain constant regardless of the total *Daphnia *density. Different size (competitor) classes respond to increasing competition in the same way.

Density dependence probably explains the discrepancy between model predictions and experimental results in [11]. A purely physiological model predicted considerably lower proportions of the total population in the hypolimnion than were found experimentally. This was explained by possible food quality effects. However, the physiological model ignores competition completely. The difference between model prediction and experimental results (at 10°C temperature gradient) is relatively small (50 % vs. 60 %) as *Daphnia *densities were in the lower part of the range covered by the present study. The value of 60 % at low densities is consistent with the results of this study (c.f. Fig. 5). At high *Daphnia *biomasses, the experimental results are about twice as large as the model predicts (80 % vs. 40 %), which shows that density dependence is an important factor in determining the vertical distribution of *Daphnia*. We are presently not able to incorporate density dependence into a model of *Daphnia *distribution. Interference competition rather than exploitative competition is important in our system with continuous food renewal, but quantitative physiological data on the effect of interference are not available. However, the ultimate goal is to construct a quantitative model of the vertical fitness distribution and compare it to the distribution adopted by the animals. Only then will it be possible to test if the assumptions of an IFD model are fulfilled.

## Conclusion

Although the assumptions of the IFD theory are very often violated, a large number of tests have found distributions that "mimic" an IFD [21]. This study has shown that density dependence is important for the distribution of *Daphnia*, hence the basic assumptions of the IFD theory are fulfilled. However, there is a need for modifications. Habitat suitability for herbivorous zooplankton is determined by food availability as well as by directed gradients of biotic (predation) and abiotic (temperature, oxygen) factors. The Ideal Free Distribution with Costs model [13] is more appropriate in this case. Further complications arise as *Daphnia *populations are size structured [36], i.e. models for unequal competitors [28] need to be applied. The experimental results conform well to the theoretical expectations considering the costs (asymmetric distribution) and differently sized *Daphnia *as unequal competitors (size specific distributions), although *Daphnia *are not omniscient and the equilibrium distribution is dynamic. This shows that the theory is rather robust.

The IFD with Costs concept can be helpful in developing a general model of vertical distribution of zooplankton. This is important as the vertical location of grazers has consequences for phytoplankton production and species composition [37,38] and, therefore, for ecosystem function. So far, the IFD with costs concept has only been applied to lake situations with a deep-water algal maximum. Although deep-water algal maxima are not rare [9], they are special cases and trade-offs will be different when algal densities are high in the epilimnion, and day-time mortality caused by fish predation [4] or UV [12] is also high. Diel vertical migrations are the logical consequence. However, predation can be considered another type of cost, and applying the IFD with Costs model may be useful for analysing vertical distributions and migrations of zooplankton under a range of environmental conditions.

## Methods

### Experimental system

The basic experimental design has been described in [11]. A large indoor mesocosm system, the Plön Plankton Towers [39], was used to test the hypothesis. These are two stainless steel columns of approximately 11.5 m height and 1 m diameter. The system can be manipulated with a vertical resolution of 50 cm and sampled through 23 vertical ports per tower. After filling the columns with filtered (10 μm) water from a nearby mesotrophic lake (Schöhsee), they were thermally stratified. As in a natural temperate lake in summer, a warm epilimnion was separated from the cool hypolimnion by a steep temperature gradient (thermocline). The same temperature gradient with the thermocline at 2.5 m depth was maintained throughout the experiment in both columns: 20°C in the epilimnion and 10°C in the hypolimnion between 2.5 and 5.1 m. The lower part of the hypolimnion (below 5.1 m) was set to 8°C (cf. Fig. 2). This created a stable layer between 2.5 and 5.1 m depth and prevented the water from being mixed into the deep layers. The diel light cycle was 14 hours light and 10 hours dark. Oxygen was monitored with an electrode. Although there was some oxygen depletion with time, the concentration never fell below 3.5 mg L^-1^.

### Preparation and sampling

To create a deep-water algal maximum a pre-cooled algal suspension was injected by tubes into the 10°C layer where it mixed within 2 hours and stayed. The green alga *Scenedesmus obliquus *Meyen, known to be good food for *Daphnia*, was cultured in 10-L jars in dilute (1:4) Z4 medium [40] under continuous light. Although the tower system cannot be kept sterile, this alga was almost the only food source for *Daphnia*. After pre-culture in 100-L containers, *Daphnia pulicaria *Forbes was introduced into the two towers in approximately equal amounts. In the absence of predation, they started building up a population.

A vertical profile of small water samples was collected every morning through sampling tubes from each tower, and the algal density was estimated using a particle counter (CASY^®^, Schärfe GmbH, Reutlingen, Germany). The estimated particle volume was converted to particulate carbon using a calibration curve. The amount of algae necessary to maintain a concentration of approximately 1.5 mg carbon L^-1 ^in the algal layer was then calculated and the missing amount replenished. This algal concentration provided saturating food conditions for *Daphnia*.

Sampling of *Daphnia *started the day after inoculation of the towers and was repeated approximately every second day (17 sampling dates in 36 days). To avoid direct light effects [17], vertical distributions of *Daphnia *were assessed at night (two hours after lights off). *Daphnia *were sampled by simultaneously pumping 48 liters of water each from sampling ports at 13 depths (between 0.1 and 10.5 m) through glass traps [39], but later only 11 ports (down to 7.5 m) were used for analysis as no *Daphnia *were found below that depth. *Daphnia *samples were preserved in sucrose formaldehyde [41]. Preserved zooplankton samples were automatically counted and sized with a bench top model Optical Plankton Counter (Focal Technologies, Dartmouth, Nova Scotia, Canada). Raw size measurements were converted to body length [42], and individuals were grouped into 4 body size classes (0.6 – 1.0 mm, 1.01 – 1.5 mm, 1.51 – 2.0 mm, 2.0 – 3.5 mm). The first two size classes comprise neonates and juveniles while adults are found in the latter two.

### Calculations and statistics

Depth distributions of *Daphnia *are given in terms of dry biomass. For each sampling port, numbers of *Daphnia *of each size class were multiplied by the mean individual mass of the size class obtained from a length-mass relationship [43] using 42 % of carbon in dry mass. Biomasses in each size class were summed to obtain the total biomass at each depth. This yielded vertical biomass profiles for each size class as well as for the total population. Vertical profiles were integrated to calculate the total biomass per tower, which was used as the independent variable for testing density dependence. For further analysis, total biomasses (dry mass per tower) of the 34 data sets (17 dates × 2 towers) were divided into five biomass groups (0.15–0.49 g, 0.5–0.79 g, 0.8–1.8 g, 1.81–3.0 g, > 3.0 g) with approximately equal (6–7) frequencies.

As the total biomass varied between dates, vertical distributions had to be analyzed as percentages of total biomass at each depth. A detailed description of the method for characterizing distribution patterns has been given in [11,17]. Biomass percentages at each sampling port were subjected to a principal components analysis (PCA) based on a variance-covariance matrix. The results of the PCA are linear combinations (principal components, PCs) of the original dependent variables (percentages at different ports). PCs represent different aspects of the distribution in terms of contrasts between the ports. Depending on the amount of variation explained by them, the first few PCs can be used to describe the main aspects of the *Daphnia *distribution, i.e. to analyze the shape of the distribution.

After testing for normality and homogeneity of variances, the factor scores of the PCs explaining most of the variation (> 90 %) were subjected to a GLM ANOVA with the estimation of the main effects (biomass group, size class, tower) and the two-way interactions between them. The ANOVA identified the experimental factors significantly related to different aspects (PCs) of the distribution, i.e. it showed which factors affected the shape of the distribution. Although the populations within a tower were sampled repeatedly, the samples were considered independent for two reasons. First, the dependent variable was the distribution. The distribution is dynamic, i.e. individuals swim up and down in the water column [16]. Hence, the population had two complete light cycles to redistribute between samplings. Second, populations were growing in numbers. New individuals were born into the population continuously while old ones died. Consequently, the population consisted in part of different individuals at each sampling date. PCA and ANOVA were carried out using the NCSS^® ^statistical package [44].

The median depth of the *Daphnia *distribution and the proportion of the total biomass dwelling in the algal maximum (between 2.5 and 5.1 m) were related to total biomass in order to get a more quantitative description of the effect of *Daphnia *density. To avoid the use of percentages in the statistics, a linear regression analysis was performed with the ungrouped, log-transformed absolute *Daphnia *biomass in the algal maximum versus the total biomass per tower. The null hypothesis was that the slope of the regression was one, i.e. there was no density dependence of the proportion in the algal maximum. Linear regressions were calculated for the sum of all, as well as for the individual, *Daphnia *size classes, and slopes and elevations were compared to test whether different size classes showed different responses to *Daphnia *density (independent variable). Regression analyses were perfomed with Statistix^® ^7.0, Analytical Software, Tallahassee, U.S.A

## Authors' contributions

W. L. designed and carried out the experiment, participated in data collection, performed data analyses, and wrote the manuscript.

## Figures and Tables

**Table 1:** Principal Components Analysis of the vertical biomass distribution of *Daphnia*. The eigenvectors of the first three principal components (PC1–PC3) account for more than 95 % of the total variation. Bold numbers depict major contrasts. The bottom line shows the cumulative percentage of the total variation explained by an individual PC.

**Sampling Port**	**Depth (m)**	**PC1**	**PC2**	**PC3**
P1	0.1	0.140	**-0.304**	-0.069
P3	1.2	0.111	**-0.331**	0.132
P4	1.6	0.104	**-0.315**	0.136
P5	2.1	0.137	-0.270	0.236
P6	2.5	**0.649**	**0.671**	-0.019
P7	3.0	**-0.535**	0.375	**0.636**
P8	3.5	**-0.460**	0.187	**-0.505**
P10	4.6	-0.137	0.005	**-0.493**
P12	5.5	-0.007	-0.011	-0.038
P14	6.5	-0.002	-0.007	-0.017
Cumulative Variance (%)		51.1	91.8	95.7

**Table 2:** Results of a GLM ANOVA on factor scores of PC1 and PC2 with estimates of the main effects of *Daphnia *biomass (Mass), size class (Size) and experimental tower, and the two-way interactions between these.

**Factor**	**d.f.**	**MS**	**F**	**p**
**PC1**				

Mass	4, 92	14.99	55.59	<0.001
Size	3, 92	6.66	24.69	<0.001
Tower	1, 92	0.92	3.42	0.068
Mass × Size	12, 92	0.74	2.74	0.003
Mass × Tower	4, 92	0.51	1.88	0.121
Size × Tower	3, 92	0.16	0.59	0.168
**PC2**				

Mass	4, 92	0.71	1.89	0.119
Size	3, 92	18.66	49.96	<0.001
Tower	1, 92	9.74	26.07	<0.001
Mass × Size	12, 92	0.15	0.40	0.960
Mass × Tower	4, 92	2.06	5.51	<0.001
Size × Tower	3, 92	1.11	2.97	0.035

**Table 3:** Linear regression analysis of the effect of total *Daphnia *biomass (B_tot_) on the biomass (B_max_) dwelling in the algal maximum (cf. Fig. 6). Comparison of the different *Daphnia *size classes. Model: log(B_max_) = b·log(B_tot_) + a.

**Size class**	**slope**	**intercept**	**R^2^**	**p**
1	1.180	-0.548	0.916	<0.001
2	1.252	-0.365	0.955	<0.001
3	1.230	-0.240	0.979	<0.001
4	1.209	-0.208	0.984	<0.001
total	1.214	-0.244	0.985	<0.001
**Comparison of regressions**		**d.f.**	**F**	**p**
Difference of slopes		3, 124	0.45	0.719
Difference of elevations		3, 127	70.43	<0.001

## References

[B1] Lampert W, Sommer U (1997). Limnoecology.

[B2] Lampert W (1993). Ultimate causes of diel vertical migration of zooplankton: new evidence for the predator avoidance hypothesis. Arch Hydrobiol Beih Ergebn Limnol.

[B3] Johnsen GH, Jakobsen PJ (1987). The effect of food limitation on vertical migration in *Daphnia longispina*. Limnol Oceanogr.

[B4] Leibold MA (1990). Resources and predators can affect the vertical distributions of zooplankton. Limnol Oceanogr.

[B5] Dini ML, Carpenter SR (1992). Fish predators, food availability and diel vertical migration in *Daphnia*. J Plankton Res.

[B6] Kerfoot WC, Rankin MA (1985). Adaptive value of vertical migration: comments on the predation hypothesis and some alternatives. Migration: Mechanisms and adaptive significance Contributions to Marine Sciences.

[B7] Gliwicz ZM, Pijanowska J (1988). Effect of predation and resource depth distribution on vertical migration of zooplankton. Bull Mar Sci.

[B8] Fee EJ (1976). The vertical and seasonal distribution of chlorophyll in lakes of the Experimental Lakes Area, northwestern Ontario: Implications for primary production estimates. Limnol Oceanogr.

[B9] Barbiero RP, Tuchman ML (2001). Results from the U.S. EPA's biological open water surveillance program of the Laurentian Great Lakes: II. Deep chlorophyll maxima. J Great Lakes Res.

[B10] Winder M, Spaak P, Mooij WM (2004). Trade-offs in *Daphnia *habitat selection. Ecology.

[B11] Lampert W, McCauley E, Manly B (2003). Trade-offs in the vertical distribution of zooplankton: ideal free distribution with costs?. Proc R Soc Lond [B].

[B12] Williamson CE, Sanders RW, Moeller RE, Stutzman PL (1996). Utilization of subsurface food resources for zooplankton reproduction: Implications for diel vertical migration theory. Limnol Oceanogr.

[B13] Tyler JA, Gilliam JF (1995). Ideal free distributions of stream fish: a model and test with minnows, *Rhinicthys atratulus*. Ecology.

[B14] Fretwell SD (1972). Populations in a seasonal environment.

[B15] Kessler K, Lampert W (2004). Fitness optimization in *Daphnia *in a trade-off between food and temperature. Oecologia.

[B16] Lampert W, Grey J (2003). Exploitation of a deep-water algal maximum by *Daphnia *: a stable-isotope tracer study. Hydrobiologia.

[B17] Kessler K, Lampert W (2004). Depth distribution of *Daphnia *in response to a deep-water algal maximum: the effect of body size and temperature gradient. Freshwat Biol.

[B18] Larsson P (1997). Ideal free distribution in *Daphnia*? Are daphnids able to consider both the food patch quality and the position of competitors?. Hydrobiologia.

[B19] Hutchinson GE (1967). A treatise on limnology II Introduction to lake biology and the limnoplankton.

[B20] Fretwell SD, Lucas HJ (1970). On territorial behavior and other factors influencing habitat distribution in birds. Acta Biotheor.

[B21] Tregenza T (1995). Building on the Ideal Free Distribution. Adv Ecol Res.

[B22] Jakobsen PJ, Johnsen GH (1987). Behavioural response of the water flea *Daphnia pulex *to a gradient in food concentration. Animal Behaviour.

[B23] Shenbrot G, Krasnov B (2000). Habitat selection along an environmental gradient: Theoretical models with an example of Negev Desert rodents. Evol Ecol Res.

[B24] Hayward RS, Gallup DN (1976). Feeding, filtering and assimilation in *Daphnia schoedleri *as affected by environmental conditions. Arch Hydrobiol.

[B25] Helgen JC (1987). Feeding rate inhibition in crowded *Daphnia pulex*. Hydrobiologia.

[B26] Lampert W (1987). Feeding and nutrition in *Daphnia*. Mem Ist ital Idrobiol.

[B27] Dawidowicz P, Loose CJ (1992). Cost of swimming by *Daphnia *during diel vertical migration. Limnol Oceanogr.

[B28] Sutherland WJ, Parker GA, Sibly RM, Smith RH (1985). Distribution of unequal competitors. Behavioural Ecology Ecological consequences of adaptive behaviour.

[B29] Milinski M (1988). Games fish play: Making decisions as a social forager. Trends Ecol Evol.

[B30] Gliwicz ZM (1990). Food thresholds and body size in cladocerans. Nature.

[B31] Kreutzer C, Lampert W (1999). Exploitative competition in differently sized *Daphnia *species: A mechanistic explanation. Ecology.

[B32] Hu SS, Tessier AJ (1995). Seasonal succession and the strength of intra- and interspecific competition in a *Daphnia *assemblage. Ecology.

[B33] Primicerio (2003). Size-dependent habitat choice in *Daphnia galeata *Sars and size-structured interactions among zooplankton in a subarctic lake (lake Lombola, Norway). Aquat Ecol.

[B34] Leibold MA, Tessier AJ (1991). Contrasting patterns of body size for *Daphnia *species that segregate by habitat. Oecologia.

[B35] Kessler K (2004). Distribution of *Daphnia *in a trade-off between food and temperature: individual habitat choice and time allocation. Freshwat Biol.

[B36] Gilliam JF, Fraser DF, Ebenman B, Persson L (1988). Resource depletion and habitat segregation by competitors under predation hazard. Size-structured populations Ecology and evolution.

[B37] Lampert W (1992). Zooplankton vertical migrations: implications for phytoplankton-zooplankton interactions. Arch Hydrobiol Beih Ergebn Limnol.

[B38] Reichwaldt ES, Wolf ID, Stibor H (2004). The effect of different zooplankton grazing patterns resulting from diel vertical migration on phytoplankton growth and composition: a laboratory experiment. Oecologia.

[B39] Lampert W, Loose CJ (1992). Plankton towers: Bridging the gap between laboratory and field experiments. Arch Hydrobiol.

[B40] Zehnder AA, Gorham PR (1960). Factors influencing the growth of *Microcystis aeruginosa *Kütz. emend. Elenk. Can J Microbiol.

[B41] Haney JF, Hall DJ (1973). Sugar coated *Daphnia *: A preservation technique for Cladocera. Limnol Oceanogr.

[B42] Kessler K, Lampert W (2003). Counting and sizing preserved *Daphnia *with the Optical Plankton Counter. Arch Hydrobiol.

[B43] Lampert W (1977). Studies on the carbon balance of *Daphnia pulex *de Geer as related to environmental conditions. II. The dependence of carbon assimilation on animal size, temperature, food concentration and diet species. Arch Hydrobiol Suppl.

[B44] Hines J (2000). NCSS 2000.

